# Genomic ancestry and adaptive signatures in the indigenous Hetian cattle from Xinjiang Province of China revealed by whole-genome sequencing

**DOI:** 10.1186/s12864-025-12346-7

**Published:** 2025-11-26

**Authors:** Xuewei Liu, Tianyong Liu, Yihua Wang, Hong Dong, Fuqiang Li, Xingshan Qi, Yongming Luo, Yi Jiang, Zulfiqar Ahmed, Chuzhao Lei, Xiang Guo

**Affiliations:** 1 Key Laboratory of Animal Genetics, Breeding and Reproduction of Shaanxi Province, College of Animal Science and Technology, Northwest A&F University, Yangling, 712100 China; 2 Shanxi Provincial Breeding Sheep Development Corporation Ltd, Jiexiu, 032000 China; 3 Department of Oncology, Taiyuan Central Hospital, Taiyuan, 030000 China; 4 Academy of Animal Sciences, Xinjiang Institute of Animal Science, Wulumuqi, 830000 China; 5 Hunan Tianhua Industrial Corporation Ltd, Lianyuan, 417100 China; 6 Animal Huabandry Bureau in Biyang County, Biyang, 463700 China; 7 Department of Livestock and Poultry Production, Faculty of Veterinary and Animal Sciences, University of Poonch Rawalakot, Muzaffarabad, Azad Jammu and Kashmir 12350 Pakistan; 8 Shanxi Bethune Hospital, Shanxi Academy of Medical Sciences, Tongji Shanxi Hospital, Third Hospital of Shanxi Medical University, Taiyuan, 030000 China

**Keywords:** Adaptive evolution, Cattle domestication, Genomic diversity, Hetian cattle, Indicine–taurine admixture, Local ancestry inference, Missense mutation, Selective sweep analysis

## Abstract

**Background:**

Cattle domestication and subsequent breed formation have profoundly shaped agricultural economies and ecological adaptation worldwide. Among these, Chinese indigenous breeds exhibit extensive phenotypic diversity driven by complex admixture histories. Hetian cattle, a native population from the arid Xinjiang Province of China, possess superior traits including drought tolerance and disease resistance. Despite their ecological and agricultural importance, the genomic architecture and adaptive mechanisms underpinning these traits remain poorly characterized.

**Result:**

To address this gap, we performed whole-genome resequencing of 20 Hetian cattle and integrated comparative analyses with 162 globally representative cattle genomes. We assessed genomic diversity, population structure, and local ancestry using a combination of principal component analysis, admixture modelling, and neighbor-joining phylogenies. Ancestry inference revealed a nearly equal taurine (49.99%) and indicine (50.01%) genetic composition, tracing to an admixture event approximately 38 generations ago. Selection signature analyses using CLR, iHS, and nucleotide diversity metrics identified genomic regions under positive selection associated with immunity (e.g., *SLAMF1*, *CD84*), high-altitude adaptation (*AGBL4*, *ALX3*), and drought resistance (*HNRNPK*, *XYLT1*, *ADPGK*). Two missense mutations (rs208626726 and rs134151223) within candidate genes may contribute to the physiological resilience of Hetian cattle.

**Conclusion:**

This study elucidates the genetic basis of local adaptation in Hetian cattle through comprehensive genomic characterization. The identification of key adaptive loci provides valuable insights into the evolutionary history and environmental resilience of this population. These findings contribute to the conservation genomics of Chinese native cattle and inform molecular breeding strategies aimed at improving adaptation and productivity under climate-stressed agroecosystems.

**Supplementary Information:**

The online version contains supplementary material available at 10.1186/s12864-025-12346-7.

## Introduction

Cattle (*Bos taurus* and *Bos indicus*) are among the most economically and culturally significant domesticated animals, playing pivotal roles in agriculture, transportation, and human sustenance worldwide. Since their domestication approximately 10,000 years ago, two primary subspecies—the humpless taurine (*Bos taurus*) and the humped indicine (*Bos indicus*)—have undergone extensive divergence, shaped by environmental pressures, human selection, and geographical barriers [1–4]. These evolutionary forces have contributed to the emergence of distinct cattle populations adapted to a broad spectrum of ecological niches, with China recognized as a global hotspot for indigenous bovine diversity, comprising 55 native breeds with region-specific traits and high adaptive potential [5–7].

The Hetian cattle, an indigenous population from the southern region of Xinjiang Province in China, exemplify adaptive excellence in extreme environments. Located at the confluence of the Pamir Plateau, Tianshan, Kunlun, and Karakoram Mountain ranges, the Hetian region is characterized by a hyper-arid desert climate and limited ecological carrying capacity. Despite such constraints, Hetian cattle thrive due to their robust drought tolerance, and disease resistance [8], making them a vital genetic resource for local communities [8]. However, the population’s limited population size—estimated at approximately 3,000 individuals—and geographic isolation have rendered it vulnerable to genetic erosion, highlighting the urgency for systematic genomic evaluation and conservation planning.

Advances in next-generation sequencing and the reduced cost of whole-genome sequencing (WGS) have revolutionized livestock genomics, enabling detailed analyses of population structure, genomic diversity, and selection signatures at unprecedented resolution [4, 9, 10]. Prior genome-wide studies have revealed the presence of hybrid zones across China where indicine and taurine lineages intermingle due to historical migrations and environmental selection [5, 6, 11]. Yet, the Hetian cattle remain understudied in this context. Their unique geographical location at the intersection of ancient migration routes suggests a potentially complex genomic ancestry, which remains to be elucidated through comprehensive genomic analyses.

To address this critical gap, we undertook WGS of Hetian cattle and integrated comparative genomic approaches to: (1) characterize their population structure and ancestral composition; (2) identify genome-wide patterns of diversity, linkage disequilibrium, and inbreeding; and (3) detect selection signatures associated with environmental adaptation. We applied ancestry deconvolution and selection scan methods to reveal key genomic regions derived from indicine and taurine lineages, shedding light on adaptive traits such as drought tolerance, disease resistance, and reproductive fitness.

By illuminating the genomic foundations of Hetian cattle, this study contributes to a deeper understanding of bovine adaptation under arid, high-stress environments. More broadly, it underscores the value of indigenous populations in global efforts towards climate-resilient livestock production and biodiversity conservation. The insights derived from this research provide a scientific basis for the preservation and genetic enhancement of native cattle, aligning with sustainable agriculture strategies in the face of escalating climate variability.

## Materials and methods

### Sample collection and whole-genome sequencing

We collected 20 Hetian cattle from a farm in Hetian Prefecture, Xinjiang Province. Ear tissue samples were collected by professional veterinarians. The collection site was disinfected immediately after sampling. No anesthesia or euthanasia was used during the collection, and the animals remained healthy after sampling. (Table S1). Genomic DNA was extracted using the standard phenol–chloroform protocol [12]. The integrity of the DNA was confirmed by 1% agarose gel electrophoresis, which showed clear, high-molecular-weight bands with minimal smearing, indicating minimal degradation (Supplementary Fig. 1). Sequencing libraries with an average insert size of 350 bp were prepared for each sample, and 150 bp paired-end sequencing was conducted on the Illumina NovaSeq platform at Novogene Bioinformatics Institute, Beijing, China. To provide comparative genomic context, we retrieved raw whole-genome sequencing reads (in FASTQ format) for 162 publicly available samples representing five major continental cattle groups were included: European taurine (Angus, Jersey and Simmental cattle *n* = 39), East Asian taurine (Hanwoo and Mishima cattle *n* = 23), Eurasian taurine (Altay, Qaidam, Kazakh, Mongolian *n* = 57), Indian indicine (Achai, Bhagnari, Cholistani, Dajal, Dhanni, Gabrali, Hisar Haryana, Gir, Lohani, Red Sindhi, Nelore, SriLanka, Hariana, Sahiwal, Nelore and Tharparkar cattle *n* = 27) and Chinese indicine (LeiqiongWenshan, Wannan, Guangfeng and Ji’an cattle *n* = 16) (Table S1). The selection of these specific breeds was based on the following rationale: first, to encompass the major historical lineages of cattle domestication (indicine and taurine) and their key geographic distributions; second, to include populations from the putative ancestral sources of Chinese indigenous cattle while avoiding overrepresentation of highly derived commercial breeds; and third, to provide sufficient genetic diversity to robustly resolve population structure and ancestry patterns in the admixed Hetian cattle population. This strategic selection minimizes potential biogeographic biases in comparative analyses. The inclusion of raw sequencing data was critical to ensure comparability. By reprocessing all public data through our identical bioinformatic pipeline, we eliminated potential biases arising from differences in alignment and variant calling methods across studies. To further guarantee data quality, we only included samples with a minimum average sequencing depth of 10× and a mapping rate greater than 95% after realignment.

### Read alignment and variant calling

Raw sequencing reads were first subjected to quality control. Adapters and low-quality bases(Phred score < 20) were trimmed using Trimmomatic v0.39.The resulting high-quality clean reads were then aligned to the *Bos taurus* reference genome assembly ARS-UCD1.2 (GCF_002263795.1) using BWA-MEM v0.7.13-r1126 with default parameters [13]. This choice of reference genome ensures maximum consistency and comparability with the vast majority of published cattle genomic studies, which have also utilized this version [4–6]. Duplicate reads were marked and removed using Picard Tools v2.18.14 (http://broadinstitute.github.io/picard). Variant calling followed GATK v3.8 Best Practices [14]: base quality score recalibration was performed using the “BaseRecalibrator” module, followed by variant calling with “HaplotypeCaller,” “GenotypeGVCFs,” and “SelectVariants.” Variants were filtered using “VariantFiltration” with the following thresholds: QD < 2.0, FS > 60.0, MQ < 40.0, MQRankSum < –12.5, ReadPosRankSum < –8.0, and SOR > 3.0. These thresholds were selected based on GATK Best Practices [14] and prior studies in cattle genomic [15]. Variants with mean depth < 1/3 × or > 3 × the average were excluded. Functional annotation of variants was conducted using ANNOVAR, with a transcript FASTA database constructed from the *Bos taurus* reference GFF file (GCF_002263795.1) [16]. Insertions and deletions (indels) and structural variants were not considered in this study due to the specific focus on SNP-based analyses for population genetics.

### Population structure and phylogenetic analyses

To assess population stratification, SNPs were pruned for linkage disequilibrium using PLINK v1.9 with the parameter –indep-pairwise 50 5 0.2 [17]. The r^2^ threshold of 0.2 ensures that only one SNP from a set of variants in high linkage disequilibrium (r^2^ > 0.2) is retained, effectively removing redundancies while preserving sufficient genome-wide markers for robust structural analysis [4]. Principal component analysis (PCA) was conducted with smartPCA in EIGENSOFT v5.0 [18] and all 182 samples were included in the PCA without prior outlier removal. Individual kinship coefficients were estimated using KING [19]. ADMIXTURE v1.3 [20] was used to infer ancestry proportions, with K values ranging from 2 to 7. We ran analyses with K (the number of ancestral populations) ranging from 2 to 10. The optimal K value was identified by plotting the cross-validation (CV) error for each K and selecting the value where the CV error was minimized, we identified the optimal K values as K = 2 and K = 4(Supplementary Fig. 2). A matrix of pairwise Hamming distances was generated using PLINK and used to construct a neighbour-joining (NJ) tree in MEGA v10.0 [21], which was visualized in iTOL [22].

#### Genetic diversity, inbreeding, and linkage disequilibrium

Nucleotide diversity (π) was estimated using VCFtools in 50-kb sliding windows with 50-kb step sizes [23]. Linkage disequilibrium (LD) decay was calculated using PopLDdecay with default settings [24]. Runs of homozygosity (ROH) were identified with PLINK using the –homozyg command with the following criteria: window size of 50 SNPs, minimum SNP density –homozyg-density 50, maximum 3 heterozygous SNPs per window, and maximum 5 missing calls. ROH lengths were binned into four categories: 0.5–1 Mb, 1–2 Mb, 2–4 Mb, and > 4 Mb. Inbreeding coefficients (F) were estimated as F = 1 − Ho/He, where Ho and He​ are observed and expected heterozygosity, respectively. All plots were generated using R (http://www.r-project.org) [25].

### Local ancestry inference

Admixture timing was inferred using ALDER [26] under a single-pulse model. Local ancestry assignment was performed using LOTER [27], with South Asian indicine and East Asian taurine populations as reference panels. Ancestry-specific segments ≥ 1,000 bp with frequency ≥ 0.7 and *P* < 0.01 (Z-test) were retained. Excessive ancestry segments were mapped using the RIdeogram package in R [28], and functional enrichment of genes within these regions was conducted via KOBAS v3.0 [6, 29].

### Selective sweep detection within Hetian cattle

Selective sweep signals in Hetian cattle were detected using a multi-faceted analytical framework incorporating three complementary approaches. First, the Composite Likelihood Ratio (CLR) test was applied to genome-wide SNP data using SweepFinder2, with calculations performed in 50-kb non-overlapping windows to identify deviations from the neutral site frequency spectrum suggestive of selective sweeps [30]. Second, nucleotide diversity (θπ) was estimated using VCFtools in sliding windows of 50 kb with a step size of 20 kb to pinpoint genomic regions exhibiting reduced genetic variability, a hallmark of recent selection events [2, 23]. Third, the integrated haplotype score (iHS), which detects extended haplotype homozygosity around a focal SNP relative to its ancestral state, was computed using Selscan v2.0 after phasing and imputation of genotype data with Beagle [31, 32]. Genomic windows ranked within the top 1% of empirical *P* values by at least two of these methods were designated as candidate regions under positive selection, thereby enhancing the robustness and reliability of sweep detection.

### Cross-population selection analysis

To identify regions under differential selection, pairwise *F*_ST_​ values were calculated using VCFtools in 50-kb windows with 20-kb steps [23]. XP-EHH statistics were computed to compare Hetian cattle against South Asian indicine and East Asian taurine groups. The XP-EHH analysis was similarly conducted using 50-kb windows with a 20-kb step size. Regions in the top 1% of both metrics were retained. Di statistics were used to further validate high-differentiation regions. Candidate genes were intersected with LOTER-assigned ancestry segments to determine origin. Tajima’s D was calculated for these genes using VCFtools. Haplotype block structures and LD heatmaps were visualized using LDBlockShow [6, 25, 33].

### Functional annotation and mutation analysis

Gene Ontology (GO) and KEGG pathway enrichment for candidate genes were performed using KOBAS v3.0 on the *B. taurus* genome [34]. Missense mutations within selective sweep regions were annotated using ANNOVAR and allele frequencies were estimated across Hetian cattle, South Asian indicine, and East Asian taurine populations.

## Results

### Genome sequencing and variant detection

**Fig. 1:**
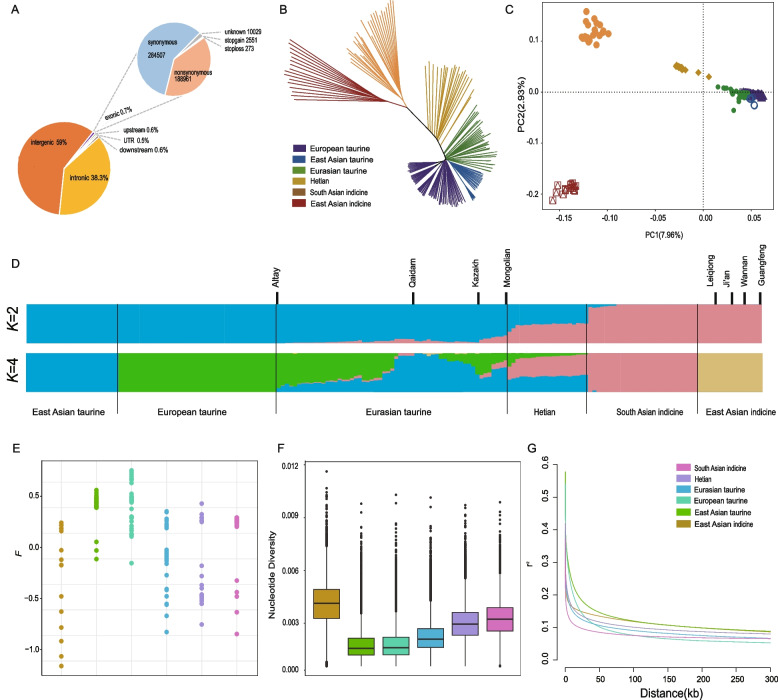
**A** Functional classification of the detected SNPs. **B** Neighbor-joining tree of the relationships in these populations. **C** Ancestry component analysis of these cattle breeds using ADMIXTURE with K = 2 and K = 4. **D** Inbreeding coefficients of each population. **E** nucleotide diversity in each population (**F**) LD decay curves of each population

Whole-genome resequencing of 20 Hetian cattle yielded an average coverage of ~ 12.6 × with a mean mapping rate of 99.69% across 182 samples, including publicly available genomes (Table S1). Variant calling and annotation identified approximately 25 million biallelic SNPs. The majority of SNPs were located in intergenic (59%) and intronic regions (38.3%), while only 0.8% resided within exonic regions, including 188,961 nonsynonymous and 284,507 synonymous variants (Fig. 1A; Table S2).

### Population structure and ancestry composition

Principal component analysis (PCA) revealed clear separation between indicine and taurine populations along the first principal component (7.96% variance), with Hetian cattle occupying an intermediate position between South Asian indicine and East Asian taurine breeds. The second component (2.93% variance) distinguished regional substructure within each lineage (Fig. 1C). ADMIXTURE analysis at K = 2 indicated near-equal proportions of taurine and indicine ancestry in Hetian cattle, while K = 4 resolved a more refined composition: South Asian indicine (50.01%), East Asian taurine (39.98%), European taurine (10.01%), and negligible South Asian indicine (< 0.01%) contributions (Fig. 1D). The NJ tree supported the admixed status of Hetian cattle, clustering them between the indicine and taurine lineages (Fig. 1B).

### Genetic diversity, ROH, inbreeding, and linkage disequilibrium

Runs of homozygosity (ROH) analysis revealed that European taurine cattle displayed the highest number and cumulative length of ROHs, while indicine cattle showed the lowest. Hetian cattle exhibited intermediate ROH values, consistent with their hybrid ancestry (Supplementary Fig. 3). Inbreeding coefficients derived from ROHs indicated moderate inbreeding in Hetian cattle (mean F = 0.044), This level of inbreeding is consistent with that reported in other admixed Chinese indigenous cattle breeds, such as Jiaxian Red cattle (F: ~ 0.0025—0.005) lower than European taurine breeds (> 0.8), but higher than South Asian indicine (–0.2767) and East Asian indicine (–0.3245) (Fig. 1E).

Nucleotide diversity (π) analysis showed that East Asian indicine had the highest diversity, followed by South Asian indicine, Hetian cattle, East Asian taurine, Eurasian taurine, and European taurine (Fig. 1F). Linkage disequilibrium (LD) decay analysis demonstrated that East Asian indicine had higher LD decay than South Asian indicine (Fig. 1G) [5]

### Admixture timing and local ancestry inference

**Fig. 2:**
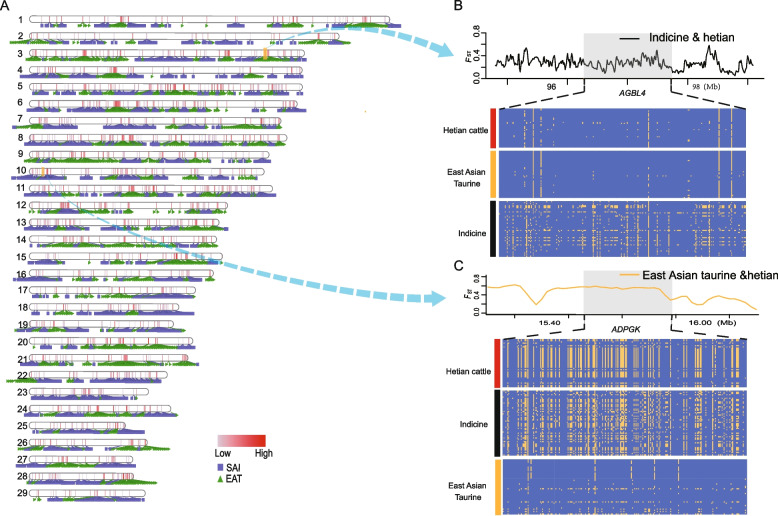
**A** Distribution of the local segments with proportions of South Asian indicine and East Asian taurine ancestries. **B** Pairwise* F*_ST_, Tajima's D value, haplotype patterns heatmap and average taurine ancestry (%) of *AGBL4* gene region. **C** Pairwise *F*_ST_, Tajima's *D* value, haplotype patterns heatmap and average taurine ancestry (%) of *ADPGK* gene region. The KEGG pathways from the enrichment analysis of genes with excessive South Asian indicine proportions

Using ALDER, the admixture event in Hetian cattle was dated to approximately 38.68 ± 9.67 generations ago, The inferred admixture time was converted to years by assuming a cattle generation interval of 5 years [26, 35], resulting in an estimated admixture event around 193.4 ± 48.35 years ago (approximately the 1820s-1830s CE) [26]. (Supplementary Fig. 4). Local ancestry inference with LOTER, using South Asian indicine and East Asian taurine as reference panels, identified 3,815 and 2,996 high-frequency ancestry-specific segments from the respective lineages (*P* < 0.01; Fig. 2A; Table S3 and S4). Excessive South Asian indicine segments were enriched for KEGG pathways including Apelin, Wnt, Hippo, Calcium, and Chemokine signalling (Table S6). In contrast, East Asian taurine segments were enriched for Ras, Cytokine–cytokine receptor interaction, Hippo, and Axon guidance pathways (Supplementary Fig. 5 and Table S5). Two representative genes—*AGBL4* and *ADPGK*—were identified as high-frequency segments with high *F*_ST_ values and distinct haplotype patterns indicative of taurine and indicine origins, respectively (Fig. 2B–C).

### Selective sweep detection

**Fig. 3:**
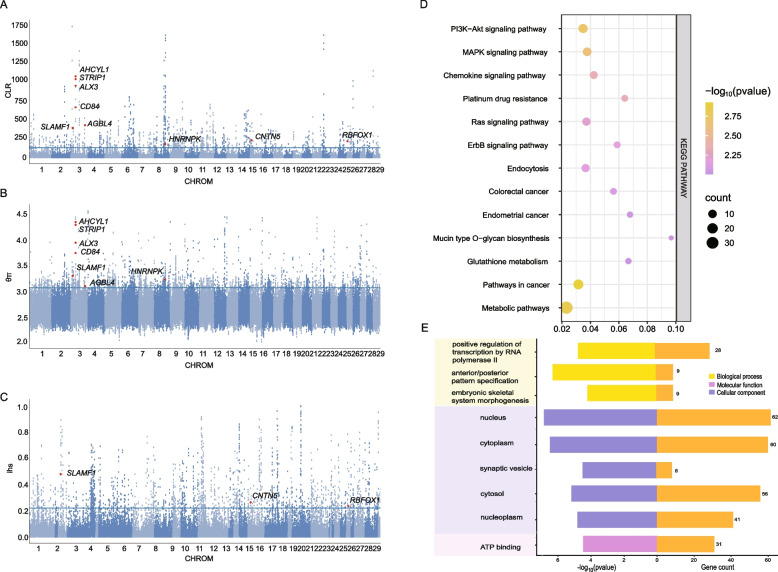
Manhattan plot of selective sweeps by (**A**) the composite likelihood ratio (CLR), **B **nucleotide diversity analysis (θπ) and **C** integrated haplotype score (iHS) in hetian cattle. **D **The KEGG pathways from the enrichment analysis of candidate genes. **E** The GO pathways from the enrichment analysis of candidate genes

Selective sweep analyses identified genomic regions under positive selection in Hetian cattle using θπ, iHS, and CLR methods. Candidate regions were defined as overlapping the top 1% empirical *P* values from at least two of these approaches (Supplementary Fig. 6; Fig. 3A-C and Table S6). Functional enrichment of 402 candidate genes highlighted pathways involved in immunity, environmental response, and development, including PI3K-Akt, Ras, MAPK, and Chemokine signalling (Fig. 3D–E; Table S7). GO terms included regulation of transcription, embryonic skeletal morphogenesis, and pattern specification.

### Cross-population selection analysis

**Fig. 4:**
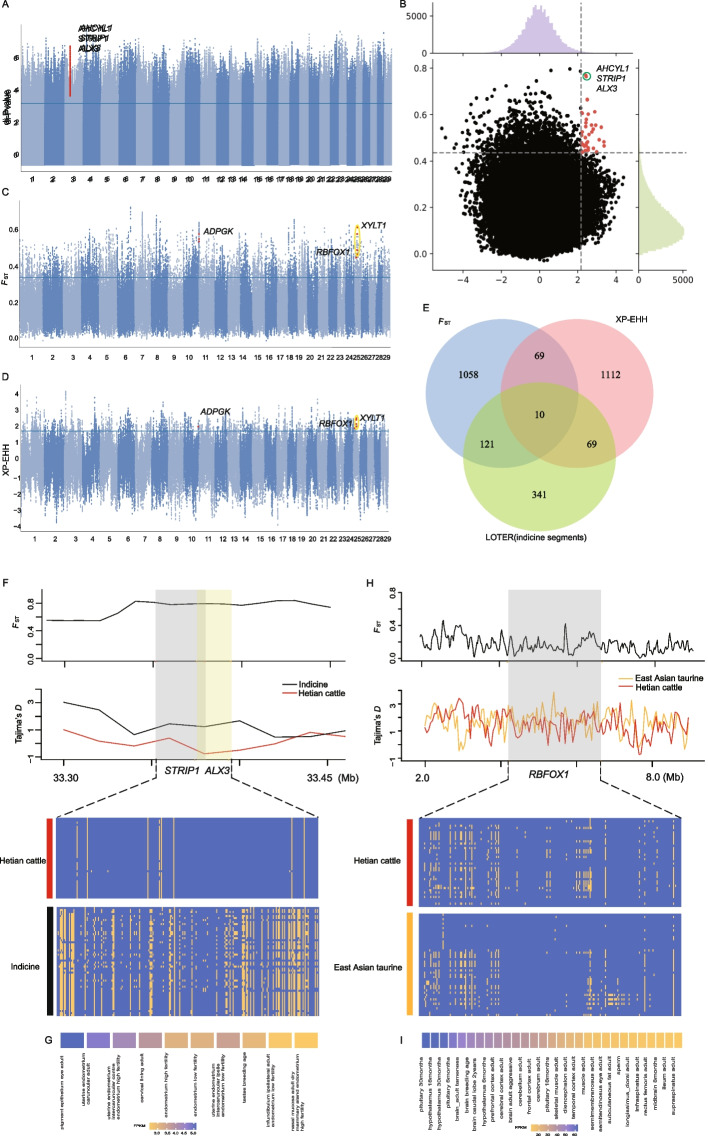
**A** Di-HT are the top 0.1% Di-SNPs from the HT versus other populations. **B** Distribution of the *F*_ST_ (y axis) and XP-EHH (y axis) between hetian and South Asian indicine cattle. Manhattan plot of selective sweeps by (**C**) *F*_ST_ and (**D**) XP-EHH methods between hetian cattle and East Asian taurine cattle. **E** venn plot of genes annotated to South Asian indicine cattle segments in loter and genes annotated to *F*_ST_ and XP-EHH. **F** Pairwise *F*_ST_, Tajima's D value, haplotype patterns heatmap of *STRIP1* and *ALX3* gene region. **H** Pairwise FST, Tajima's D value, haplotype patterns heatmap of *STRIP1* and *RBFOX1* gene region. **G** Gene expression of *ALX3* in different cattle tissues (http://animal.nwsuaf.edu.cn/code/index.php/RGD). **I** Gene expression of RBFOX1 in different cattle tissues (http://animal.nwsuaf.edu.cn/code/index.php/RGD)

Pairwise *F*_ST_ and XP-EHH comparisons between Hetian cattle and South Asian indicine cattle identified 88 overlapping candidate genes under divergent selection (Fig. 4A-B Table S8). Among these, the region on BTA3:33.36–33.41 Mb harboured *AHCYL1*, *STRIP1*, and *ALX3*, each showing high *F*_ST_, low Tajima’s *D*, and distinct haplotypes (Fig. 4F). Expression analysis from the Ruminant Genome Database revealed tissue-specific expression of *ALX3* in the reproductive system (Fig. 4G).

Similarly, comparison with East Asian taurine cattle yielded 79 genes identified by both *F*_ST_ and XP-EHH (Fig. 4C-E Table S9). Key candidates included *XYLT1*, *RBFOX1*, and *ADPGK*, associated with bone growth, neural regulation, and reproductive traits, respectively. These genes were confirmed to originate from indicine segments, with haplotype and Tajima’s D analysis supporting selective fixation (Fig. 4H). Expression analysis of candidate genes was conducted using RNA-seq data from the Ruminant Genome Database (RGD, version 2.0, http://animal.nwsuaf.edu.cn/code/index.php/RGD) Only samples with RNA integrity number (RIN) > 7 and mapping rate > 90% were included in the analysis. Transcripts per million (TPM) values were used for comparative expression profiling across tissues..(Fig. 4G; 4I).

### Linkage disequilibrium and mutation analysis

**Fig. 5:**
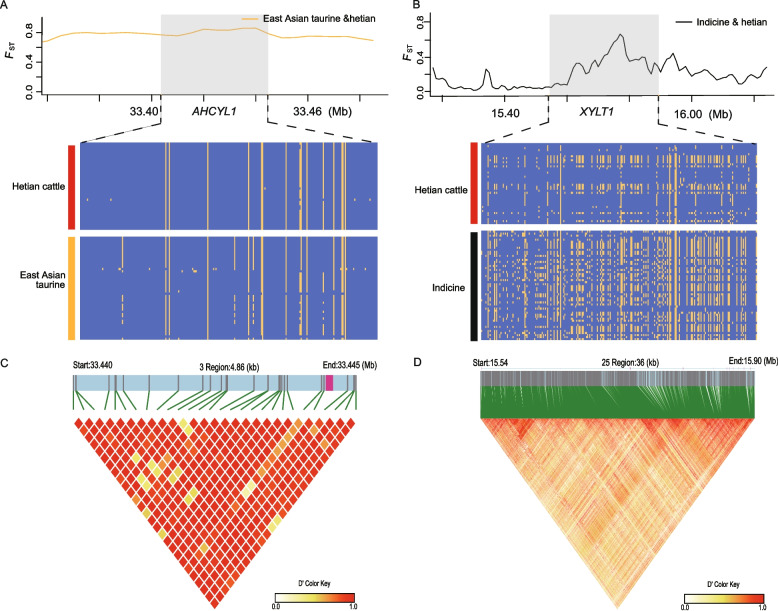
**A** Pairwise *F*_ST_ and haplotype patterns of *AHCYL1* gene region. **B** Pairwise *F*_ST_ and haplotype patterns of *XYLT1* gene region. **C** LD plot of SNPs. LD values (D′) between two loci are detailed in boxes (D′ = 0–1). D′ = 1 indicates perfect disequilibrium (BTA 33.440—33.445). **D** LD plot of SNPs. LD values (D′) between two loci are detailed in boxes (D′ = 0–1). D′ = 1 indicates perfect disequilibrium (BTA 19.546—19.578)

LD analysis revealed strong haplotype conservation in regions containing *AHCYL1* (East Asian taurine origin) and *XYLT1* (South Asian indicine origin), with elevated LD (D′ = 1) indicating selective sweeps (Fig. 5A–D). Two missense mutations, rs208626726 in *ADPGK* and rs134151223 in *CD84*, were identified within selective sweep regions. Allele frequency analysis showed that rs208626726 was elevated in both Hetian and South Asian indicine cattle, while rs134151223 was lower in these populations compared to East Asian taurine cattle (Supplementary Fig. 7).

## Discussion

Understanding the genetic architecture of indigenous livestock breeds is critical for conserving biodiversity and developing sustainable breeding strategies, particularly under the accelerating pressures of climate change [36]. This study investigated the genomic landscape of Hetian cattle, a local population from Xinjiang Province, China, known for its adaptation to arid environments and valued for meat quality and disease resistance [8]. By integrating whole-genome resequencing with comprehensive population genomic analyses, we have clarified the population’s genetic composition, evolutionary history, and molecular signatures of adaptation.

The observed SNP distribution reflects typical patterns of mammalian genomic variation, with the majority of variants located in non-coding regions, and a smaller but functionally important fraction residing in coding exons. The identification of 188,961 nonsynonymous SNPs provides a valuable reservoir of potentially functional variants that may underpin the phenotypic traits unique to Hetian cattle. This depth of annotation enhances the resolution for subsequent evolutionary and functional genomic analyses [16].

This study has limitations, the most notable being the moderate sample size (*n* = 20) for whole-genome sequencing of the Hetian core population. While a larger sample size would improve the detection of rare alleles and refine estimates of genetic diversity and inbreeding, we consider our main conclusions robust for two key reasons. First, the population genetic parameters we estimated (e.g., nucleotide diversity π, inbreeding coefficient F) align with those reported for other Chinese indigenous cattle breeds based on larger samples [5]. Second, the clear ancestry patterns and strong, convergent selection signals detected by multiple methods (CLR, θπ, iHS) are unlikely to result from limited sampling, as such pronounced genomic signals remain detectable even with moderate sample sizes when genetic drift or selection is strong. Nonetheless, future studies with expanded sampling across the Hetian region would help capture potential genetic sub-structure and further validate the selection signatures reported here.The population structure and phylogenetic analysis reveal that Hetian cattle occupy an intermediate genetic position between South Asian indicine and East Asian taurine cattle. The admixture model at *K* = 4 highlighted a substantial dual ancestry, with nearly equal contributions from taurine and indicine lineages. Notably, Hetian cattle retained a minor proportion of European taurine input, likely reflecting historic gene flow or unaccounted introgression events [5]. These findings are in agreement with the known migration history of cattle into East Asia, whereby indicine breeds spread eastward from the Indus Valley and taurine breeds entered China via West Asia [1–3, 11]. Hetian cattle exhibit a nearly balanced genetic ancestry (50.01% indicine, 49.99% taurine), reflecting the region's history as a Silk Road hub that facilitated livestock exchange. This stands in stark contrast to the recently reported Indian Vrindavani crossbred cattle, which exhibits a genome predominantly derived from European taurine ancestry (~ 67.3%) [37]. This disparity underscores their distinct breeding histories and selection objectives: Vrindavani cattle were developed through targeted crossbreeding for high milk yield, whereas Hetian cattle represent an indigenous breed shaped by long-term natural and artificial selection to arid and high-altitude environments in Northwest China. The high indicine proportion provides crucial drought tolerance traits essential for arid summers, while the significant East Asian taurine ancestry contributes genetic adaptations to cold winters and potentially influences meat quality.

The ROH and inbreeding analyses positioned Hetian cattle as a moderately inbred population, higher than indicine breeds but lower than intensively selected European taurine breeds. This suggests the absence of recent strong artificial selection, consistent with their status as a locally conserved breed. Importantly, the observed intermediate nucleotide diversity and LD decay further support the admixed origin and relatively large effective population size of this population [23–25].

Although using South Asian indicine and East Asian taurine populations as reference panels offers a reasonable approximation for deciphering the ancestry of Hetian cattle, several limitations must be considered. Modern reference populations may not accurately represent the historical ancestral groups that actually contributed to the Hetian genome, due to genetic drift and selection that have occurred in all populations since the time of admixture. As a result, ancestry from lineages that have diverged significantly from contemporary references may be underestimated. Admixture dating via ALDER indicated that the primary hybridization event occurred approximately 38 generations ago (~ 190–200 years), temporally aligning with historical pastoral expansions and cross-breeding practices in western China [26]. The ALDER analysis indicates that the main admixture event in Hetian cattle occurred about 38.68 ± 9.67 generations ago (∼193 ± 48 years). Although this timing interestingly overlaps with historical increases in Silk Road trade and migration, several sources of uncertainty should be noted. The relatively wide confidence interval may be due to: (1) the assumption of a single admixture pulse, which oversimplifies what was likely a more continuous process; (2) genetic drift after admixture, which distorts ancestry signals; (3) potential inaccuracy in the assumed 5-year generation interval; and (4) inherent noise in genetic data. Thus, while this estimate offers a useful temporal reference, it should be seen as an approximate period when major gene flow began, rather than an exact date. More complex demographic models may improve dating accuracy in the future.

This relatively recent admixture implies that Hetian cattle have retained significant genomic segments from both ancestral sources, providing a model to investigate adaptive introgression. Local ancestry deconvolution using LOTER confirmed the presence of numerous taurine- and indicine-specific segments. Genes from excessive South Asian indicine regions were enriched in pathways related to immune response, stress signaling, and cellular regulation (e.g., Wnt, Apelin, and Chemokine signaling), reflecting their probable contribution to environmental resilience in arid settings [27–29].

Conversely, taurine-derived segments were associated with developmental and neurobiological processes, including Ras signaling and axon guidance. The *AGBL4* gene, previously implicated in high-altitude adaptation in yak [38], was identified within a taurine-originated segment, while *ADPGK*, associated with metabolism and immune modulation [39–44], was located within an indicine-originated region. These findings underscore the functional asymmetry of introgressed segments and support the hypothesis that Hetian cattle benefit from complementary adaptive traits inherited from both lineages.

The application of multiple complementary selection detection methods (CLR, θπ, iHS) provided robust identification of genomic regions under positive selection. Enrichment analyses of 402 candidate genes revealed functional categories critical for survival in harsh environments, including *MAPK, PI3K-Akt*, and Chemokine signaling—pathways linked to inflammation, stress response, and reproductive function [45, 46]. Importantly, *SLAMF1*, *CD84*, and *CNTN5* were among immune-related genes identified as under selection, consistent with prior findings in other native cattle and livestock populations [4, 47, 48]. While our integrated approach using CLR, θπ and iHS robustly identifies genomic regions with extreme patterns indicative of selection, it is important to note that these methods can be confounded by demographic history, such as population bottlenecks or expansions. However, we employed several strategies to increase our confidence in the results: first, the convergence of signals from multiple independent statistics (CLR, θπ, iHS) in the same genomic region reduces the likelihood of a false positive driven solely by demography. Second, the functional enrichment of candidate genes in biologically relevant pathways (e.g., immunity, environmental response) provides additional biological plausibility that the observed signals are adaptive.

Cross-population selection comparisons using *F*_ST_ and XP-EHH detected differentiation in genomic regions [25], between Hetian cattle and both South Asian indicine and East Asian taurine breeds. The region encompassing *STRIP1*, *ALX3*, and *AHCYL1* showed strong signatures of selection. *STRIP1* has been implicated in mesoderm migration and axis development [49], while *AHCYL1* plays roles in cell cycle regulation and programmed cell death [50–52]. Expression data further indicated elevated *ALX3* activity in reproductive tissues, suggesting that these taurine-derived genes may influence reproductive fitness under selection in Hetian cattle.

Within indicine-derived regions, strong differentiation was observed at *XYLT1*, *RBFOX1*, and *ADPGK*. *XYLT1* is linked to bone development and has been implicated in desert adaptation in jerboas [53, 54], while *RBFOX1* has demonstrated roles in neurotransmission and behavioral traits in cattle [55]. These genes may represent genomic targets for resilience in thermally and nutritionally challenging environments typical of the Hetian region.

Functional enrichment analysis of candidate genes provides critical insights into the biological processes and systemic mechanisms underlying local adaptation, revealing coordinated network-level responses. Key signaling pathways significantly enriched in this study include PI3K-Akt, Ras, MAPK, chemokine, Apelin, Wnt, Hippo, and calcium signaling, highlighting their important roles in coordinating adaptive responses to environmental stress. Among these, the PI3K-Akt [56] and MAPK pathways are particularly crucial for regulating cell survival, proliferation, and metabolic adaptation, while chemokine and Apelin signaling contribute to immunomodulation and vascular homeostasis [57, 58]. The convergence of Wnt [59, 60], Hippo [61], and Ras [62] pathways further underscores their involvement in developmental processes and tissue maintenance. This multipathway synergy, refined through historical admixture and sustained environmental pressures, demonstrates a highly integrated genetic architecture that enhances the adaptability of Hetian cattle to arid and high-altitude conditions.

To further confirm the functional importance of the identified key candidate genes, we investigated their overlap and previous associations with known quantitative trait loci (QTLs). Of note, the overlap of SLAMF1 and CD84 genes with somatic cell count and mastitis resistance QTL highlights their potential role in enhancing immune response [63]; The co mapping of XYLT1 gene related to skeletal development and drought resistance of desert species with QTLs for height and weight [54, 64], indicates its contribution to the structural adaptation of Hotan cattle in arid environments; The AGBL4 gene derived from yellow cattle, which is related to high-altitude adaptation, is located within a QTL interval that has been clearly reported to be associated with bovine blood oxygen saturation and pulmonary artery pressure [65]. The pleiotropy of these genes highlights their importance in climate adaptation and overall productivity breeding, strongly supporting our hypothesis that the selection signals we detected are indeed associated with adaptive phenotype variations.

The identification of two missense mutations—rs208626726 in *ADPGK* and rs134151223 in *CD84*—with divergent allele frequencies between Hetian and reference populations provides preliminary evidence of potentially functional adaptive variants. The fixation patterns, LD structure, and ancestral origins of these alleles support their candidacy as targets of recent selection, meriting further functional validation. To assess their potential functional impact, we performed in silico prediction using PolyPhen-2. The mutation rs134151223 (p.Val128Met in CD84) was predicted to be 'benign' (score: 0.040). While this suggests that this specific amino acid change may not be highly deleterious to protein structure, its location within a strong selective sweep region, its differential allele frequency between populations, and its occurrence in the immunologically relevant CD84 gene still make it a compelling candidate for association with the disease-resistant phenotype.

To sum up, this study presents a comprehensive genomic characterization of Hetian cattle, revealing their recent admixture origin, moderate genetic diversity, and unique mosaic ancestry shaped by local adaptation. The identification of candidate genes and mutations associated with immunity, stress response, and development provides valuable insights into adaptive mechanisms in arid environments. These findings have direct implications for conservation genomics, informing targeted breeding programs aimed at enhancing productivity and climate resilience in Chinese native cattle. We can design strategic crossbreeding schemes that leverage the complementary strengths of the ancestral genomes. For instance, crossing Hetian cattle (which carry indicine resilience) with high-yielding taurine breeds could be optimized by using our ancestry-specific markers to select crossbred offspring that retain the key indicine adaptive segments while introgressing productivity traits. Future work should priorities functional validation of key adaptive loci and the integration of transcriptomic and phenotypic data to further elucidate the genotype–phenotype relationships underlying environmental adaptation.

## Conclusion

This study provides a detailed genomic dissection of Hetian cattle, revealing a recent and near-equal admixture between South Asian indicine and East Asian taurine lineages. Population structure, diversity indices, and local ancestry analyses demonstrate a mosaic genome shaped by adaptive introgression. Selective sweep and cross-population comparisons identified candidate genes associated with immunity (*SLAMF1*, *CD84*), environmental resilience (*HNRNPK*, *XYLT1*), high-altitude adaptation (*AGBL4*), and reproduction (*ALX3*, *ADPGK*), alongside two putative adaptive missense mutations. By integrating local ancestry inference (LOTER) with cross-population selection scans (XP-EHH, *F*_ST_), we could not only identify selected genes but also assign their ancestral origin—a crucial step forward in understanding how admixed populations harness variation from divergent lineages to adapt. These findings enhance understanding of local adaptation in admixed cattle and offer a genomic foundation for conservation and targeted breeding strategies aimed at improving resilience and productivity in arid environments. In the future, we will prioritize in-depth research on gene regions associated with core adaptive traits, such as drought resistance and immunity. Additionally, key adaptive mechanisms including high-altitude adaptation and reproductive gene regions will also receive significant attention.

## Supplementary Information

Supplementary Material 1

## Data Availability

The datasets supporting the conclusions of this article are available in the NCBI Database Sequence Read Archive, BioProject number PRJNA1202368.

## Abbreviations

SNPs: Equine chromosome
WAS: Whole-genome sequencing
NJ: Neighghbor-Joining
PCA: Principal Component Analysis
PC1: First principal component
PC2: Second principal component
ROH: Runs of homozygosity
LD: Linkage disequilibrium
