# Supplementary figures and images for: Genomic ancestry and adaptive signatures in the indigenous Hetian cattle from Xinjiang Province of China revealed by whole-genome sequencing
Source: BMC Genomics. 2025 Nov 26;26:1148. doi: 10.1186/s12864-025-12346-7 (PMC12751780; doi:10.1186/s12864-025-12346-7)

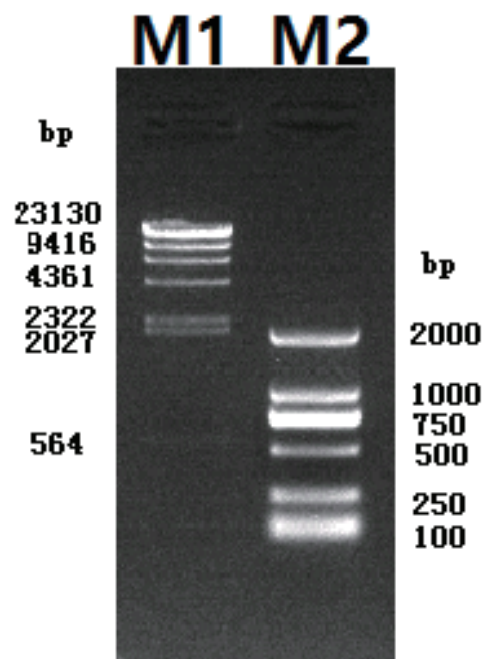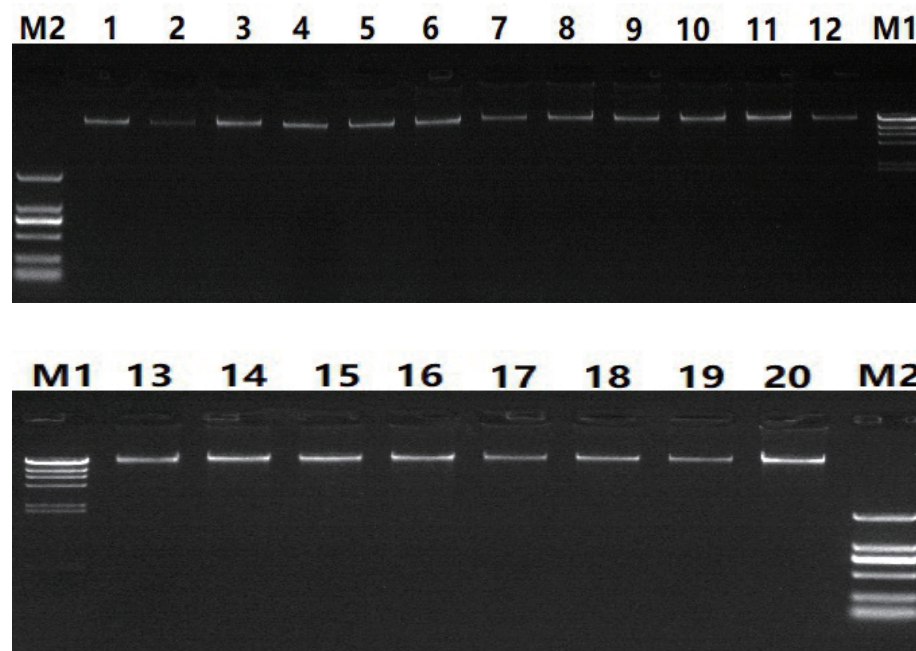

Supplement: Supplementary file 1 — Supplementary Material 1 [file 12864_2025_12346_MOESM1_ESM.zip › Supplementary materials/Supplementary Figure 1.pdf]

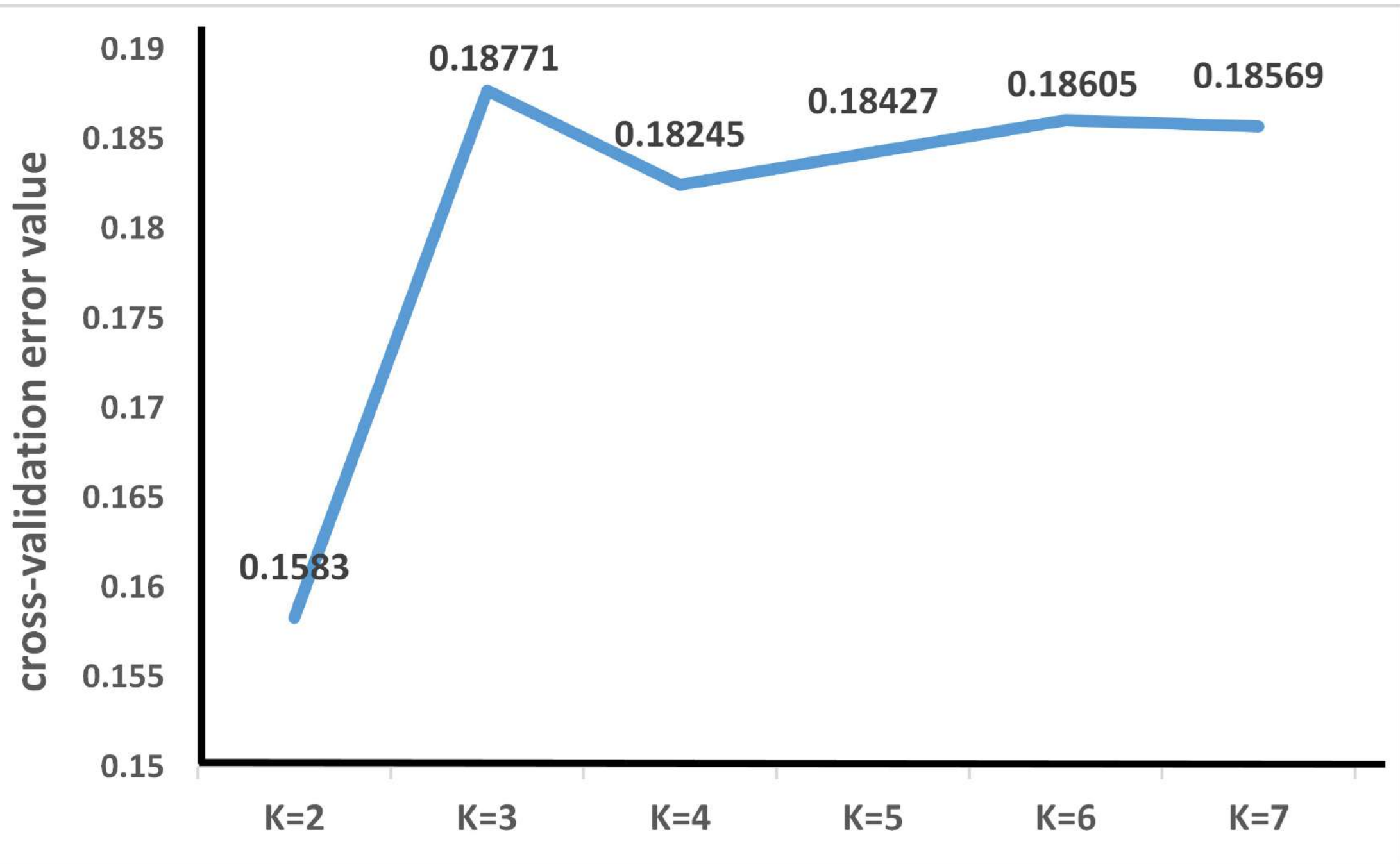

Supplement: Supplementary file 1 — Supplementary Material 1 [file 12864_2025_12346_MOESM1_ESM.zip › Supplementary materials/Supplementary Figure 2.pdf]

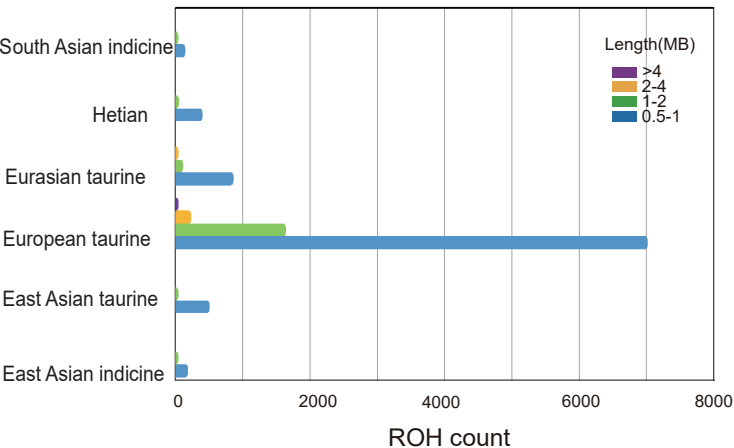

Supplement: Supplementary file 1 — Supplementary Material 1 [file 12864_2025_12346_MOESM1_ESM.zip › Supplementary materials/Supplementary Figure 3.pdf]

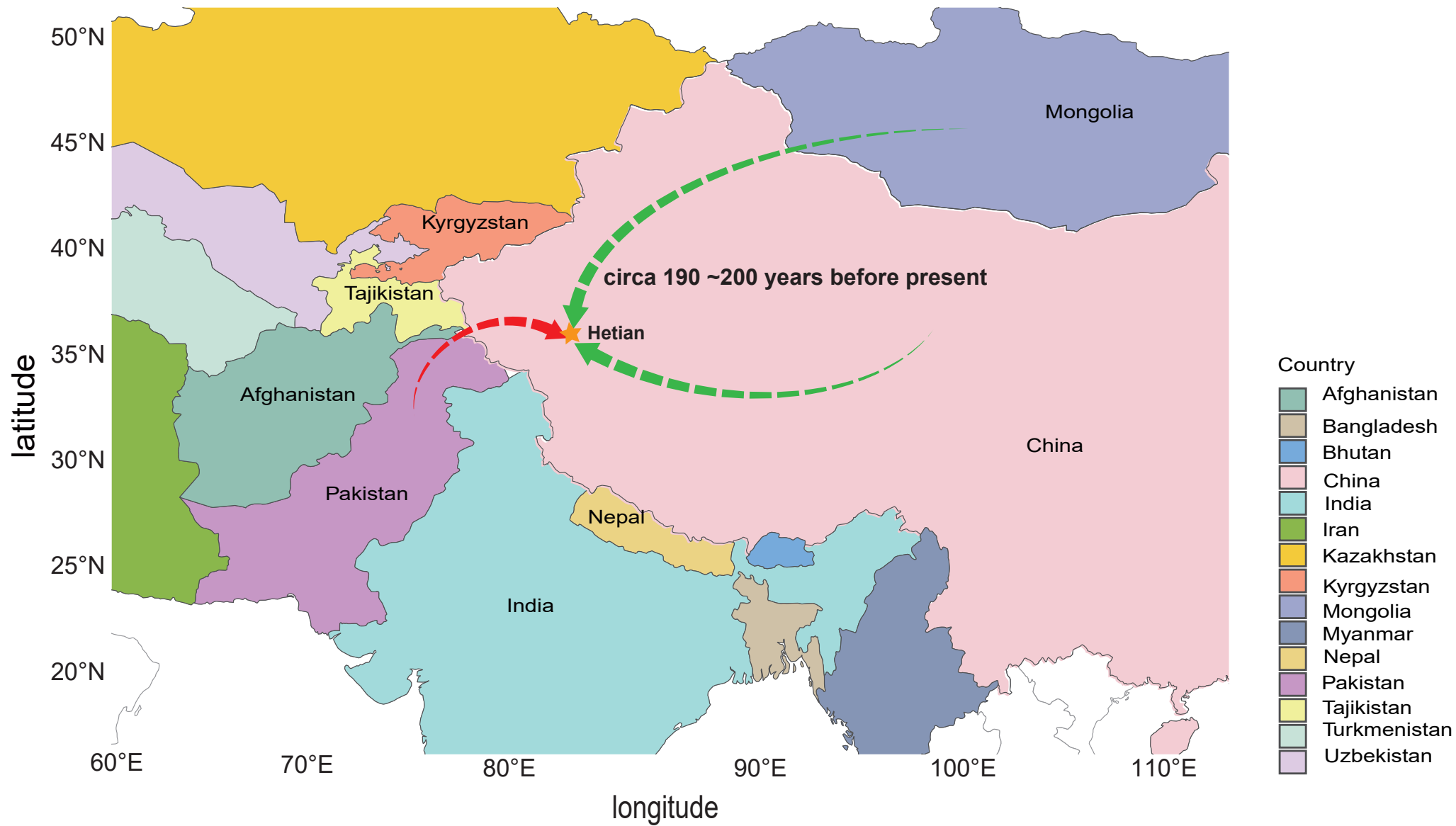

Supplement: Supplementary file 1 — Supplementary Material 1 [file 12864_2025_12346_MOESM1_ESM.zip › Supplementary materials/Supplementary Figure 4.pdf]

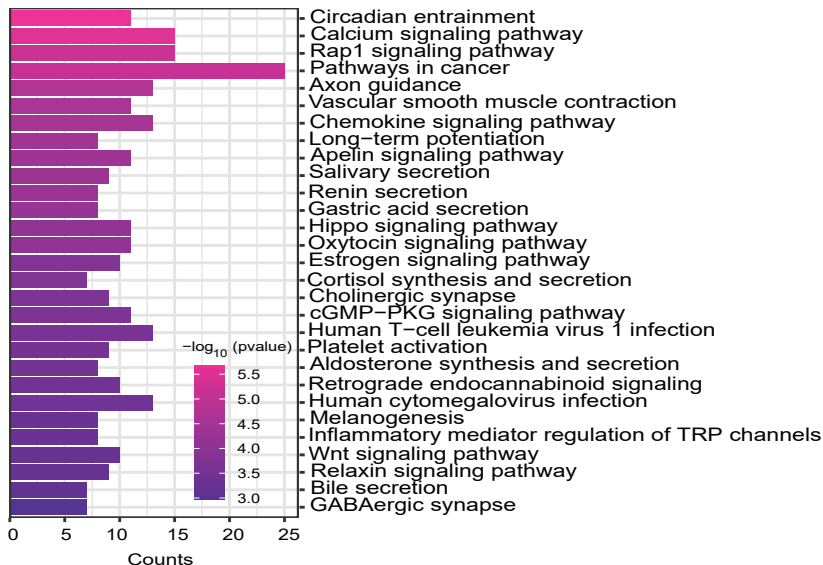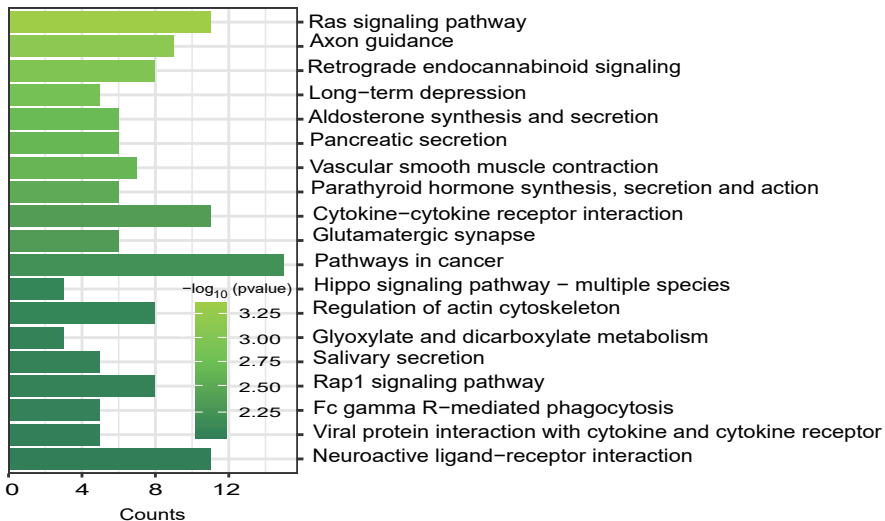

Supplement: Supplementary file 1 — Supplementary Material 1 [file 12864_2025_12346_MOESM1_ESM.zip › Supplementary materials/Supplementary Figure 5.pdf]

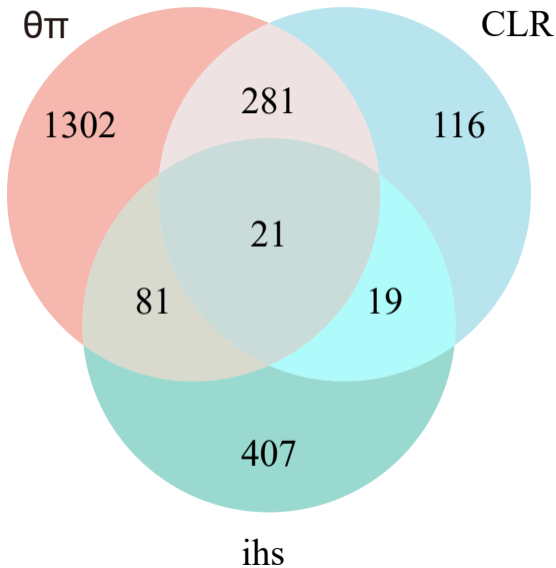

Supplement: Supplementary file 1 — Supplementary Material 1 [file 12864_2025_12346_MOESM1_ESM.zip › Supplementary materials/Supplementary Figure 6.pdf]

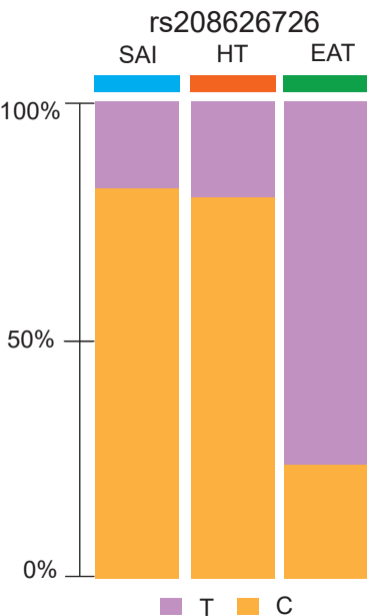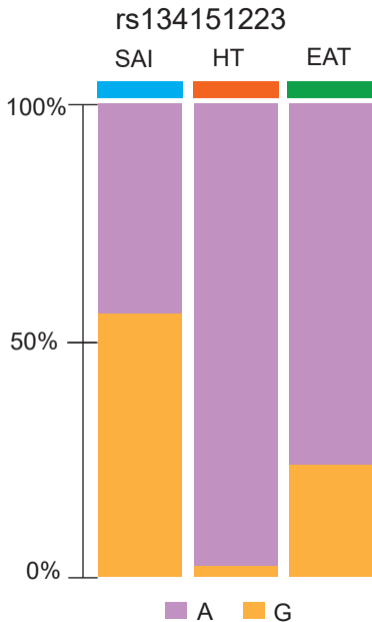

Supplement: Supplementary file 1 — Supplementary Material 1 [file 12864_2025_12346_MOESM1_ESM.zip › Supplementary materials/Supplementary Figure 7.pdf]
