# Supplementary material for: Genomic ancestry and adaptive signatures in the indigenous Hetian cattle from Xinjiang Province of China revealed by whole-genome sequencing
Source: BMC Genomics. 2025 Nov 26;26:1148. doi: 10.1186/s12864-025-12346-7 (PMC12751780; doi:10.1186/s12864-025-12346-7)
Supplement: Supplementary file 1 — Supplementary Material 1 [file 12864_2025_12346_MOESM1_ESM.zip › Supplementary materials/supplementary figure.doc]

**Supplementary Fig. 1** Agarose Gel of DNA

**Supplementary Fig. 2** cross-validation error value

**Supplementary Fig. 3** The distribution of total number of ROH across chromosomes.

**Supplementary Fig. 4** Schematic illustration of the inferred the admixture dating of hetian cattle using alder.

**Supplementary Fig. 5** The KEGG pathways from the enrichment analysis of genes with excessive East Asian taurine proportions.

**Supplementary Fig. 6** venn plot of genes annotated by the composite likelihood ratio (CLR), nucleotide diversity analysis (θπ) and integrated haplotype score (iHS) in hetian cattle.

**Supplementary Fig. 7** Allele frequency of the two missense SNPs in three group.
